# The angiogenic factor midkine is regulated by dexamethasone and retinoic acid during alveolarization and in alveolar epithelial cells

**DOI:** 10.1186/1465-9921-10-77

**Published:** 2009-08-21

**Authors:** Huayan Zhang, Samuel J Garber, Zheng Cui, Joseph P Foley, Gopi S Mohan, Minesh Jobanputra, Feige Kaplan, Neil B Sweezey, Linda W Gonzales, Rashmin C Savani

**Affiliations:** 1 Division of Neonatology, Department of Pediatrics, Children's Hospital of Philadelphia, University of Pennsylvania School of Medicine, Philadelphia, PA, USA; 2 Departments of Human Genetics and Pediatrics, McGill University, Montreal, Canada; 3 Division of Respiratory Medicine, Departments of Pediatrics and Physiology, The Hospital for Sick Children, University of Toronto, Toronto, Canada; 4 Divisions of Pulmonary & Vascular Biology and Neonatal-Perinatal Medicine, Department of Pediatrics, University of Texas Southwestern Medical Center, Dallas, TX, USA

## Abstract

**Background:**

A precise balance exists between the actions of endogenous glucocorticoids (GC) and retinoids to promote normal lung development, in particular during alveolarization. The mechanisms controlling this balance are largely unknown, but recent evidence suggests that midkine (MK), a retinoic acid-regulated, pro-angiogenic growth factor, may function as a critical regulator. The purpose of this study was to examine regulation of MK by GC and RA during postnatal alveolar formation in rats.

**Methods:**

Newborn rats were treated with dexamethasone (DEX) and/or all-trans-retinoic acid (RA) during the first two weeks of life. Lung morphology was assessed by light microscopy and radial alveolar counts. MK mRNA and protein expression in response to different treatment were determined by Northern and Western blots. In addition, MK protein expression in cultured human alveolar type 2-like cells treated with DEX and RA was also determined.

**Results:**

Lung histology confirmed that DEX treatment inhibited and RA treatment stimulated alveolar formation, whereas concurrent administration of RA with DEX prevented the DEX effects. During normal development, MK expression was maximal during the period of alveolarization from postnatal day 5 (PN5) to PN15. DEX treatment of rat pups decreased, and RA treatment increased lung MK expression, whereas concurrent DEX+RA treatment prevented the DEX-induced decrease in MK expression. Using human alveolar type 2 (AT2)-like cells differentiated in culture, we confirmed that DEX and cAMP decreased, and RA increased MK expression.

**Conclusion:**

We conclude that MK is expressed by AT2 cells, and is differentially regulated by corticosteroid and retinoid treatment in a manner consistent with hormonal effects on alveolarization during postnatal lung development.

## Background

Lung development consists of embryonic, pseudoglandular, canalicular, saccular, and alveolar stages that define a dynamic progression from a rudimentary lung bud to a saccule with a completely developed respiratory tree. The formation of alveoli involves mesenchymal thinning and the development of crests, or secondary septae, at precise sites of the saccular wall. These crests protrude into the saccular air space, include the inner layer of the capillary bilayer, and further subdivide the saccule into subsaccules that later become mature alveoli. The end result is the formation of a complex distal airway structure with a dramatic increase in the surface area available for gas exchange. While not fully understood, the mechanisms regulating secondary septation involve several cell types including endothelial cells, myofibroblasts, and epithelial cells as well as growth factors, hormones, and environmental conditions that either inhibit or stimulate alveolar growth [1].

Lung development in humans reaches its final stage around 35 weeks of gestation, with alveolarization and microvascular maturation continuing postnatally for at least three years if not longer. Lung development in rodents matches that in humans except that alveolarization is entirely a postnatal event, occurring in the first three weeks of life [2,3]. This process is associated with decreased plasma corticosteroid concentrations [4], and administration of corticosteroids during this period inhibits alveolarization [5]. Using a neonatal rat model, Massaro and others have demonstrated the effects of dexamethasone (DEX) and all-*trans*-retinoic acid (RA) treatment on alveolar development. DEX-treated animals develop a simplified architecture with impaired secondary septation and large terminal air sacs, whereas RA-treated animals develop smaller, more numerous alveoli. DEX-induced changes are ameliorated in animals that receive concomitant DEX+RA administration [6].

In rodent models, a precise balance exists between the actions of endogenous GC and retinoids to promote normal lung development, in particular during alveolarization. The mechanisms controlling this balance are largely unknown, but recent evidence suggests that midkine (MK) may function as a critical regulator. MK, a 13 kDa heparin-binding growth factor, is a RA-responsive gene involved in numerous processes including cell migration, tumor progression, inflammation, and angiogenesis. During murine development, MK expression is widespread early in gestation and becomes restricted to specific sites by late gestation [7]. Further, in the normal developing lung, MK expression increases from PN2, peaks at PN4, and decreases thereafter [8]. In addition, MK has been implicated in mesenchymal thinning in a lung explant culture system [9]. Not affected, however, was branching morphogenesis, a process known to play a key role in the earlier pseudoglandular stage of lung development [9]. Lastly, we have previously shown that MK is upregulated in glucocorticoid receptor knockout mice, and that GC and RA differentially regulate MK in vitro [10]. Collectively, these data suggest that MK is normally decreased in late gestation, corresponding to increased GC and decreased RA signals.

The purpose of this study was to examine regulation of MK expression by GC and RA during postnatal alveolar formation in neonatal rat pups. We hypothesized that MK expression in both lungs and in isolated AT2 cells would be decreased by corticosteroids and increased by RA.

## Methods

### Reagents

Cell culture media, antibiotics and fetal calf serum were obtained from Invitrogen Inc. (Carlsbad, California). Restriction enzymes, modifying enzymes and other molecular biology reagents were purchased from Promega (Madison, WI), Roche Applied Sciences (Indianapolis, IN) and New England Biolabs Inc. (Beverly, MA). Dexamethasone and 8-bromo-cAMP were purchased from Sigma Chemical Company and ^35^S-methionine was purchased from Perkin-Elmer Inc. (Boston, MA). All other chemicals were obtained from either Sigma Chemical Company (St. Louis, MO) or Fisher Scientific Inc. (Pittsburgh, PA) unless otherwise specified. H441 and A549 cells were obtained from American Type Culture Collection (Rockville, MD).

### Fetal Lung Epithelial Cell and Fibroblast Isolation and Culture

We isolated enriched populations of epithelial cells from second trimester (14-20 wk) human fetal lung tissue obtained from Advanced Bioscience Resources, Inc. (Alameda, CA) under IRB-approved protocols of the Children's Hospital of Philadelphia (CHOP). Epithelial cells were isolated and cultured as previously described [11]. Briefly, after overnight culture as explants [12], the tissue was digested with trypsin, collagenase and DNase, and fibroblasts were removed by differential adherence as described [13]. Non-adherent cells were plated on 60 mm plastic culture dishes in Waymouth's medium containing 10% fetal calf serum. After overnight culture (d1), attached cells were cultured an additional 2 days or 4 days in 1 ml of serum-free Waymouth's medium alone (control), or with dexamethasone (DEX, 10 nM), plus 8-Br-cAMP (0.1 mM) and isobutylmethylxanthine (IBMX, 0.1 mM), a combination referred to as DCI, or with DEX or 8-Br-cAMP/isobutylmethylxanthine (cAMP) separately. In addition, cultured cells were treated with all-*trans*-retinoic acid (RA, 5 μM) with or without concomitant DEX, or with RA+cAMP, or with RA+DCI. In previous studies, we showed that DCI promotes differentiation of the isolated fetal lung epithelial cells toward a type II cell phenotype. As compared to DCI, Dex or cAMP individually induced only partial type II cell differentiation. In addition, our previous studies have established that epithelial cell purity by this procedure is 83 ± 2%, with fibroblasts as the primary contaminating cell type [14].

Fibroblasts from the same fetal lung tissue were recovered as the adherent cells during isolation/purification of undifferentiated epithelial cells, allowed to grow for 3 days, then trypsinized and plated for the hormone treatments (1 passage eliminated epithelial cells from the population). After overnight adherence, fibroblasts were cultured for 48 h in different hormone combinations (DEX or DCI with or without RA).

### Animals

All protocols were reviewed and approved by the CHOP Institutional Animal Care and Use Committee and in accordance with NIH guidelines. Timed pregnant Sprague-Dawley rats (Charles River Breeding Laboratory, Wilmington, MA), were maintained until parturition on a 12:12 h light:dark cycle with unlimited access to food (Purina Lab Diet, St. Louis, MO) and water in the Laboratory Animal Facility at CHOP.

Within 12 hours of birth, litters were adjusted to 10 pups per litter and divided into the following treatment groups: (1) Dexamethasone (DEX, American Regent Laboratories, Inc., Shirley, NY) 0.1 μg in 20 μl 0.9%NaCl [saline]) or saline alone (20 μl) subcutaneously (SQ) daily from PN1-14; (2) all-*trans*-retinoic acid (RA, Sigma-Aldrich, St. Louis, MO) 500 μg/kg in 20 μl cottonseed oil (CSO, Sigma-Aldrich, St. Louis, MO) or CSO alone (20 μl) via intraperitoneal (IP) injection daily from PN3-14; (3) DEX and RA at doses and days as above; (4) saline and CSO at doses and days above; and (5) control (same handling, no injections). The dose of DEX was based on previous literature demonstrating only mild effects on somatic growth [6]. Animals were studied at PN1, 5, 10, and 15. Because it was difficult to discern the gender of rats at birth, both males and females were studied.

### Lung Harvest

Anesthesia for all studies was attained using an intramuscular injection of a Ketamine/Xylazine (87:13 μg/kg) cocktail. The right lung was removed, snap frozen in liquid nitrogen, and stored at -80°C for future analysis. As previously described [15], the left lung was inflated to 25 cm H_2_O pressure with formalin and stored in formalin for 24 hours before switching to 70% alcohol. Water displacement was used to measure lung volume immediately after inflation with maintenance of inflation confirmed by repeat measurement 24 hours after fixation. Lungs were then processed to obtain 5-micron thick paraffin sections. For each time point, sections were stained with hematoxylin and eosin in order to examine lung architectural differences using light microscopy.

### Radial alveolar counts (RAC)

RAC were obtained to quantify alveolarization as previously described [16]. Briefly, a perpendicular line to the edge of the sample was drawn from the center of a bronchial or bronchiolar airway to either the edge of the lung or the nearest connective tissue septum or airway. A minimum of forty lines were drawn for each lung, and the number of septae intersected was counted for each line. In addition, at least three sections from several levels within the tissue block were used for each animal. RAC is a well established method to quantify alveolarization and previous investigators [17] have confirmed that forty measurements per lung are sufficient to establish a reliable morphometric assessment of alveolarization. All RAC calculations were performed using images at 40× magnification.

### Western Blot Analysis

Western blot analysis was performed using samples obtained from both rat lung tissue and cultured Type II cells using the NOVEX NuPAGE electrophoresis system (Invitrogen) with 1 mm 4-12% BisTris gels according to manufacturer's instructions. Briefly, 10 μg of lysate was loaded to each well and gels were run at 200V at 4°C for 50 min in NuPAGE MOPS SDS running buffer under reducing conditions. Proteins were transferred to nitrocellulose membrane at 30V for 60 min at room temperature. The membrane was then blocked for 1 h at room temperature with 5% nonfat dry milk in Tween/Tris-buffered saline (TTBS) (100 mM Tris base, 1.5 M NaCl adjusted to pH 7.4 with 0.1% Tween 20). The primary antibody, Midkine H-65 (Santa Cruz Biotech, Santa Cruz, CA), was then applied overnight at 4°C. On the following day, the membrane was washed with TTBS four times, for 10 min each time and a horseradish peroxidase-conjugated goat anti-rabbit secondary antibody was applied for 1 h at room temperature. Following this, the membrane was washed with TTBS followed by two 15-min washes with TBS. The blots were developed using a chemiluminescence system (Amersham Pharmacia Biotech, Piscataway, NJ). Equal loading was confirmed by stripping and immunoblotting for β-actin, which was also used to normalize the data for densitometric analysis. Specificity was also confirmed by probing the blots with normal IgG, which yielded no consistent bands (data not shown).

Semi-quantitative densitometric analysis of bands was accomplished on a Macintosh G3 Power PC computer using MacBAS version 4.2(Fujifilm) after subtraction of background density. Results were calculated as the degree of change from control values after normalization to β-actin densitometry. The results of at least five animals per condition and each time point were expressed as mean ± SEM and normalized as percent of control.

RNA Isolation Total RNA was obtained from snap-frozen tissue maintained on ice during isolation. Tissue (~250 mg wet weight) was mechanically homogenized and total RNA was isolated with RNA Stat-60 reagent (Tel-Test, Friendswood, TX). Purity was verified by measuring the ratio of absorbance at 260 nm and 280 nm. Quantity and integrity of RNA was measured using the eukaryote total RNA nano assay on an Agilent 2100 bioanalyzer (Agilent, Palo Alto, CA). Integrity was also confirmed using 1% agarose gels.

### Reverse Transcription and Quantitative Real-Time PCR

cDNA was made from total RNA using random primers with SuperScript RT-PCR kit (Invitrogen) following the manufacturer's protocol. Quantitative real-time PCR was performed to assess the induction of Tie1 mRNA as a marker of endothelial cell content in response to the hormonal treatments. Relative mRNA expression was assessed using polymerase-activated fluorescent PCR probes providing continuous message quantification during amplification (TaqMan, Applied Biosystems, Foster City, CA). Differences in gene expression were determined by comparing the number of PCR cycles required to achieve a threshold of fluorescent activity above background during the exponential phase of the reaction. Sample loading was normalized by the simultaneous amplification of GAPDH. All reactions were performed in triplicate and the average threshold cycle for the triplicate was used in all subsequent calculations. GAPDH primer/probe set and Tie 1 probe (5'-FAM fluorescent-reporter-AGCTGCCTACATCGGAGACGCACC-3') were purchased from Applied Biosystems. Tie 1 forward primer 5'-GCCCTTTTAGCCTTGGTGTGT-3', and reverse primer 5'-TTCACCCGATCCTGACTGGTA-3' were obtained from Integrated DNA Technologies, Inc. (Coralville, IA).

### Northern Blot Analysis

The membrane was prehybridized for 2 h at 65°C in hybridization solution [0.5 M sodium phosphate, pH 7.5,7% SDS, 1 mM EDTA, 1% BSA, 50 μg/ml poly(A)^+ ^RNA, and 50 μg/ml of denatured and sheared salmon sperm DNA]. Midkine cDNA probes were labeled by random priming using the Ready-To-Go Kit (Pharmacia-Upjohn) per the manufacturer's instructions and were purified with a G-50 column. The 28S oligonucleotide probe was 5'-end labeled using a 5'-end-labeling protocol (35-50 ng of 28S oligonucleotide, 2 μl of T4 polynucleotide kinase, and 50 μCi of [γ-^32^P]ATP in 1× kinase buffer) at 37°C for 1 h per the manufacturer's instructions (Promega, Madison, WI). The probe was purified with a G-25 column (Boehringer Mannheim, Indianapolis, IN). Hybridization of membranes with ^32^P-labeled probes (1 × 10^6 ^counts·min^-1^·ml^-1^) was performed for 16-18 h at 65°C. The blots were then washed with saline-sodium citrate-0.1% SDS and were developed using a PhosphorImager (Storm 840; Molecular Dynamics, Sunnyvale, CA).

Semi-quantitative densitometric analysis of bands was accomplished on a Macintosh G3 Power PC computer using MacBAS version 4.2(Fujifilm) after subtraction of background density. Results were calculated as the degree of change from control values. The results of at least five animals per condition and each time point was expressed as mean ± SEM and normalized to percent of control.

### Statistical Analysis

Statistical comparisons between groups were carried out using ANOVA with Fisher's exact test and Bonferroni correction for individual comparisons. All *p *values less than 0.05 were considered significant.

## Results

### Effects of Hormonal Manipulation on Distal Lung Architecture

**Figure 1:**
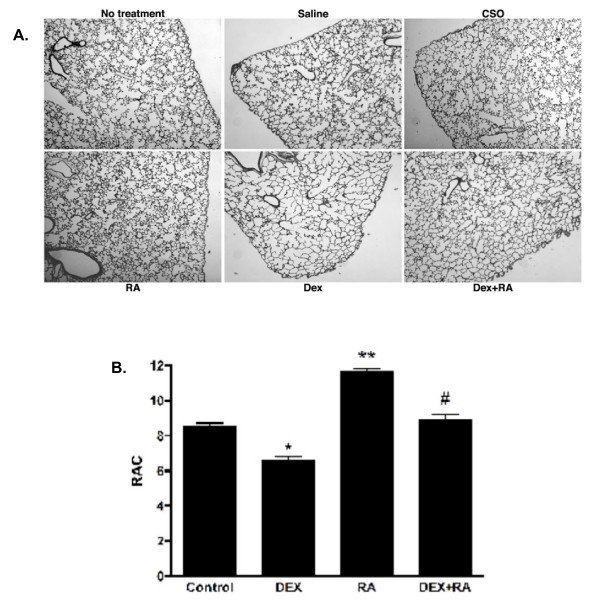
**Morphologic changes in the lung at PN15 after hormonal treatments**. (A). A simplified distal architecture was seen in DEX-treated animals. RA-treated animals had smaller and more numerous alveoli. Concomitant DEX and RA administration resulted in septation similar to that of controls. Vehicle (saline or CSO) treatment alone had no effects on lung histology. Control: same handling, no injections. DEX: Dexamethasone. RA: all-trans-retinoic acid. CSO: cottonseed oil. (B). Radial Alveolar Counts confirm the decreased septation seen with DEX treatment (*p < 0.001 DEX vs. control), the increased septation seen with RA (*p < 0.001 RA vs. control), and the resolution of DEX effects by concomitant RA administration (**p < 0.001 DEX vs. DEX+RA). Data are representative of at least 6 rats per treatment group. All images 40× magnification.

Neonatal rat pups were treated with DEX and/or RA, or appropriate controls, during the first two weeks of life as described in Methods. Representative histology and radial alveolar counts at PN15 is shown in Figure 1. At PN15, DEX-treated animals had larger, simpler distal air spaces than saline controls, with a decreased RAC as compared to control animals (*p < 0.05). These structural changes were evident as early as PN5 (data not shown, see ref. 33). RA-treated pups, on the other hand, had smaller, more numerous alveoli and higher RAC (**p < 0.05) than CSO controls as early as PN5 and up to PN15. Resolution of corticosteroid-induced changes in architecture was seen between PN10 and 15 in pups treated with concomitant DEX and RA, such that, at PN15, the lungs displayed architecture similar to that of controls and RAC were the same as controls (# p < 0.05 vs. DEX).

### Expression of Midkine and Effects of Hormonal Treatment

**Figure 2:**
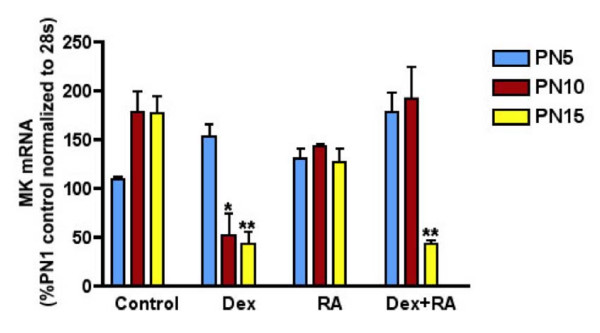
**Hormonal regulation of lung MK mRNA expression**. mRNA content expressed as percentage of PN1 control normalized to 28 s. Data are shown as mean ± SEM. DEX treatment inhibited and RA treatment had no effect on MK mRNA expression on PN10 and 15. Concomitant RA treatment was unable to restore DEX-induced decrease in MK expression at PN15 (*p < 0.01 DEX vs. control at PN10, **p < 0.001 DEX or DEX+RA vs. control at PN15).

Northern blot analysis was carried out for each treatment group at each time point studied (Figure 2). Data are shown as percent PN1 control levels. Data from the three control groups (no treatment, saline and CSO treatment) were combined since the vehicle treatments had no effect on MK mRNA expression. In control animals, MK mRNA increased between PN5 and PN10. Dexamethasone treatment had a biphasic effect, increasing MK mRNA precociously, between PN1 and PN5, and then decreasing content at PN10 and PN15. RA alone had minimal effects on the developmental pattern. However, with co-treatment, the inhibition observed with dexamethasone was delayed until PN15.

**Figure 3:**
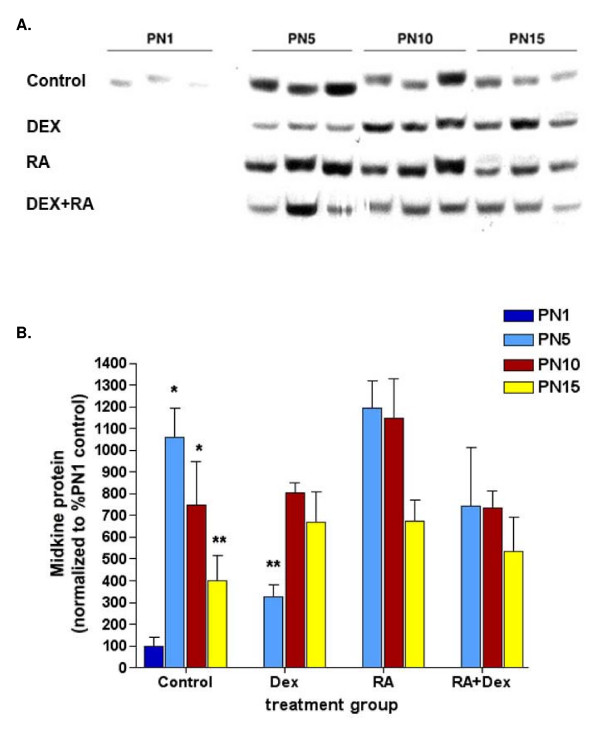
**Hormonal regulation of lung MK protein**. A) Representative Western blots of MK expression in neonatal rat lungs after various treatments. B) Densitometry analysis confirmed that, in control animals, MK protein content was highest at PN5, with a 10.5-fold induction from day 1 (*p < 0.01, n = 3), and decreased thereafter (**p = 0.02 PN5 control vs. PN15 control, n = 3/group). In contrast, MK was significantly decreased in DEX-treated lungs at PN5 with a 3-fold reduction compared to the same day control animals (**p < 0.01, n = 3). An increase in MK similar to control animals was seen at PN5, but this increase was sustained up to PN10 in RA-treated animals being 1.5 fold higher than PN10 controls. Concomitant DEX+RA treatment resulted in a return of protein levels to that of control.

A representative Western blot for MK is shown in Figure 3a with a histogram demonstrating densitometric analysis with normalization with β-actin for equal loading in Figure 3b; (β-actin blots not shown). In concordance with the known temporal expression patterns of MK, protein levels were highest in control animals at PN5, with a 10.5-fold induction from PN1, and decreasing thereafter. Dexamethasone treatment delayed the increase in MK with a 3-fold reduction (p < 0.01, n = 3) compared to control animals at PN5. Corresponding to the architecture in RA-alone treated lungs, an increase in MK similar to control animals was seen at PN5. This increase was sustained up to PN10 in RA-treated animals being 1.5 fold higher than the same day controls. Concomitant DEX+RA treatment resulted in protein levels similar to those of controls. These data confirmed that no relationship exists between steady state mRNA and protein levels for MK [8].

### Changes in Tie1 expression during hormonal treatment partially correlated with the changes in MK expression and lung morphology

**Figure 4:**
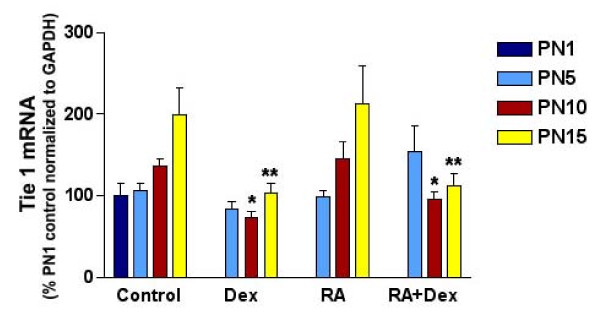
**Tie 1 mRNA expression during hormonal treatment**. mRNA content expressed as percentage of control normalized to GAPDH. Data are shown as mean ± SEM (n = 4-9 group). DEX treatment significantly decreased Tie1 expression at both 10 and 15 days (*p = 0.0006 and **p = 0.0022 respectively) as compared to same days controls. RA treatment alone did not change Tie 1 expression and also failed to restore DEX-induced decrease in Tie 1 expression (* and **p = 0.04).

MK plays a significant role in angiogenesis. We therefore wanted to test if Tie1, a marker of endothelial cells, would change during hormonal treatment and correlate with the changes in MK. Expression of Tie1 mRNA was determined by real Time RT-PCR (n = 4-9 per group). As shown in Figure 4, Tie1 expression was significantly decreased in DEX-treated animals at both PN10 and 15 compared to control (*P = 0.0006 and 0.0022 respectively). At PN5, there was a trend toward decreased Tie1 expression with DEX and increased Tie1 expression when RA was added to DEX treatment. However, this did not reach statistical significance (*p *= 0.08). RA treatment alone did not change Tie 1 expression and also failed to restore DEX-induced decrease in Tie 1 expression at PN10 and 15 (RA+DEX vs. control: **p = 0.04 at PN10 and **p = 0.01 at PN15).

### Hormonal Regulation of MK in ATII-like Cells

We next examined the expression and hormonal regulations of MK in isolated human alveolar epithelial cells and fibroblasts. We used a well-established method of alveolar epithelial cell isolation and culture. DCI promotes the differentiation of isolated undifferentiated epithelial cells towards a type II epithelial cell phenotype. In the same system, DEX or cAMP alone induces only partial differentiation. We therefore examined the effect of different hormone combinations on MK expression.

**Figure 5:**
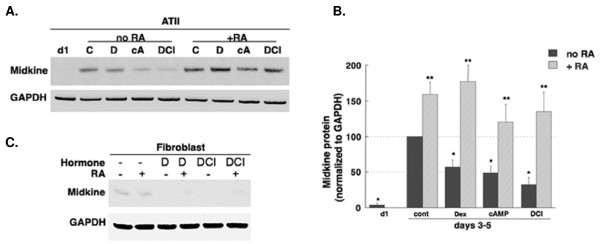
**Hormonal regulation of MK in isolated human Type II-like cells**. A) Representative western blot and B) Densitometry analysis of MK expression in human fetal alveolar epithelial cells treated with different hormone combination: Alveolar epithelial cells obtained from second trimester human fetal lung tissue treated with hormones (DEX, cAMP and IBMX, or DCI) to differentiate them into alveolar type II (ATII) cells have significantly decreased MK protein content at day 3 and day 5 as compared to controls with no treatment (*p < 0.01). However, RA treatment alone or concomitant RA treatment with hormones was associated with significant increase in MK protein expression (**p < 0.05). C) Fetal lung fibroblasts isolated from the same second trimester human fetal lung tissue were treated with DEX or DCI with/or without RA. Expression of MK was very low irrespective of treatment groups. GAPDH expression was used as a loading control.

Western blot analysis of MK regulation in Type II-like cells and lung fibroblasts are shown in Figure 5. The levels of MK protein expression with various treatments were similar on PN3 and PN5. Therefore, combined densitometry data are shown in figure 5B. MK expression increased 10-fold during cell culture without hormones or serum. Cells treated with hormones (DEX, cAMP, or DCI) had significantly decreased MK protein levels, with an apparent additive effect of GC and cAMP to repress the culture-induced increase in MK and RA eliminated the repressive effects of hormones (**p < 0.05 vs. no RA).

Fetal lung fibroblast had minimal MK expression with or without hormone treatment (Figure 5c), whereas ATII-like cells showed much more robust MK expression especially in the presence of RA. These data suggest that alveolar epithelial cells, and not fibroblasts, are the primary source of MK.

## Discussion

In the present study, we show that, in normal lungs, midkine (MK) protein content is highest at PN5, and begins to decline by PN10. This finding is in concordance with Matsuura et al. who showed a transient increase in MK expression in normal lungs between two to seven days postnatally [8]. We extend these observations to demonstrate that, in vivo, GC treatment is associated with lower and RA treatment with higher lung MK protein expression. However, in our hands, changes in steady state MK mRNA did not match MK protein expression after hormonal treatments. Hormonally driven changes in protein expression were also seen in cultured human type II-like epithelial cells, but not fibroblasts, isolated from second trimester human fetal lung tissue.

The regulation of the balance between the actions of GC and RA on lung development is largely unknown. Studies by Kaplan et al have suggested that MK might serve as a potential bridge between these two systems [18]. MK is a retinoic acid-responsive, heparin binding growth factor that promotes angiogenesis, cell growth, and cell migration [19,20]. A bimodal temporal-spatial expression pattern of MK is seen in the developing mouse lung. High levels of MK expression are observed at embryonic day (E)13-16.5 and then again at postnatal days 5-12, primarily in respiratory epithelium early in lung development and increasingly localized to lung stroma and pulmonary blood vessels postnatally [21]. However, its expression is completely absent from the adult mouse lung. These findings suggest that MK may be involved in epithelial differentiation, vascular growth and remodeling in the developing lung and is not required for regular lung maintenance.

Although MK was initially identified as a retinoic acid-responsive gene, mechanisms regulating its expression in the lung have not been fully understood. Examples of these MK regulators include thyroid transcription factor (TTF)-1 [22], and hypoxia-inducible factor (HIF)-1 [23]. Through gene array analysis of GC receptor knockout mice, Kaplan et al demonstrated that MK is dynamically regulated by both GC and retinoic acid during normal fetal lung development [10]. While these observations provided a potential mechanism for the integration of GC and retinoid effects in late gestation fetal lung development, whether GC and RA also influence MK gene expression during postnatal lung development remained unknown. In this study, we found that GC treatment induced an early suppression of MK protein expression at PN5, whereas RA treatment was associated with higher and persistent MK expression to PN10 in neonatal rats. This regulatory pattern of MK expression by GC and RA is even clearer in the isolated human fetal lung epithelial cells. Collectively, our data suggest that MK is likely differentially regulated by GC and RA from the late saccular to early alveolar stage of lung development.

Prolonged treatment with high doses of GC was widely used in immature infants with evolving bronchopulmonary dysplasia (BPD) during the 1990s. These treatments were based on the belief that such treatment was associated with less early postnatal lung inflammation and a reduction in the incidence of BPD among premature infants [24]. However, subsequent clinical trials of DEX treatment, beginning at 2-4 weeks after birth, failed to demonstrate differences in ventilation requirements or incidence of BPD, and showed toxic effects including increased risk of infection, hyperglycemia and abnormal neurodevelopmental outcome in exposed patients [25-27]. These toxic effects of high-dose steroids have also been documented in animal studies [28,29]. Further, there is evidence from rodent studies that postnatal steroid treatment also inhibits alveolarization and reduces lung growth [30]. The serum concentration of GC reaches a nadir during the period of maximum secondary septation, whether prenatal or postnatal, and increases as septation ends [4,31]. This suggests that endogenous corticosteroids might be inhibitors of septation. Indeed, our present study shows that treatment with DEX results in simplified distal lung architecture with reduced secondary septation in neonatal rats. These results are in agreement with the findings of Blanco et al [32] and our previous studies [33].

The mechanism(s) by which DEX inhibits septation is not well understood, but may be related to the inhibitory effect of GC on DNA synthesis and cell proliferation [34]. Discontinuing corticosteroids after the "critical period" of alveolarization is not followed by spontaneous septation. The process of alveolar septation requires active replication of epithelial and other cells. GC therefore might prevent septation via its ability to inhibit cell division [5,34]. In addition, this failed septation is accompanied by a reduced number of pulmonary arteries and a restricted alveolar capillary bed [35]. Our results demonstrating decreased Tie1 expression with DEX treatment further support these findings.

Several lines of evidence have indicated that retinoids might be important regulators of alveolarization. Initial evidence was provided by Brody et al. who reported that fibroblasts rich in vitamin A storage granules form a large fraction of the alveolar wall during septation [36,37]. These lipid-rich fibroblasts play a key role in producing elastin at the sites of new secondary septa [38,39]. Retinoids signal through their receptors, RARs and RXRs. Indeed, deletion or inhibition of RAR results in reduced elastin and alveolar simplification [40,41]. Studies by Massaro et al have shown that RA treatment results in increased septation in newborn rats and also induces alveoli formation in adult rats with elastase-induced emphysema [42,43]. In humans, low levels of vitamin A have been found in premature babies at risk for BPD and vitamin A supplementation reduces the incidence of BPD in these babies [44,45]. Consistent with these studies, and providing a potential mechanism by which retinoids might decrease the incidence of BPD, we show that animals receiving retinoic acid (RA) treatment had smaller and more numerous alveoli and that concomitant treatment with DEX and RA prevented the DEX-induced changes in septation.

Closely linked to the development of distal alveolar structures is the formation of a mature vascular plexus [46]. The transition from saccular to alveolar stages of lung development correlates with microvascular development and allows for close apposition of the vascular bed and airspace for efficient gas exchange to occur [44]. The molecular signals that link these two processes are not clear. However, a complex interplay of epithelial-endothelial cells is most likely required for normal lung morphogenesis. Recently, the "vascular hypothesis" of BPD [47] has proposed that inhibition of vascular growth itself may directly impair alveolarization. Several observations support the importance of vascular formation as vital for normal alveolar development. For example, treatment of neonatal rat pups with anti-angiogenic drugs, such as thalidomide, or VEGF receptor blocker is associated with a simplified distal lung architecture and decreased vascularization [48]. In addition, FGF receptor 3 and 4 double knockout mice fail to develop a mature distal lung architecture [49]. Further, decreased endothelial cell migration by blocking anti-PECAM-1 antibody or in PECAM-1 null mice is associated with disrupted alveolarization [50]. In humans, an abnormal alveolar capillary network and decreased expression of endothelial cell markers have been found in premature newborns dying with BPD [51]. The fact that GC treatment decreased MK expression both in vivo and in cultured type II lung epithelial cells, (as demonstrated by the current study), and also decreased Tie1 expression on PN10 and 15, suggests that GC might inhibit alveolarization by interfering with epithelial-endothelial communication via MK and altering normal alveolar septal vascular development. However, RA treatment had no effect on Tie1 expression and also failed to rescue the decreased Tie1 expression caused by DEX-treatment in our study. This suggests that the RA-induced enhancement in septation and the rescue of GC-induced inhibition of alveolarization may not be mediated by affecting endothelial content.

## Conclusion

In summary, we have demonstrated that MK is differentially regulated by corticosteroid and retinoid treatment during postnatal lung development, and that its expression matches the hormonal effects on alveolarization. MK may, therefore, serve as a paracrine signal that originates in the epithelium, targets pulmonary vascular cells and influences lung vascularization during the alveolar and microvascular maturation phase of lung development.

## Competing interests

The authors declare that they have no competing interests.

## Authors' contributions

HZ was responsible for part of the animal studies, performing statistical analyses, performing real-rime PCR analysis, and drafting the manuscript. SJG was responsible for some animal studies and measuring radial alveolar counts. ZC performed the Northern and Western blots for MK from the animal samples. JPF, MJ and GSM assisted in animal harvesting and injections, as well as some data analysis. FK and NBS helped conceive the study and design initial experiments. LWG was responsible for the determination of MK expression in alveolar type II cells and fibroblasts. RCS conceived the study, participated in its design and coordination, and helped to write and revise the manuscript. All authors read and approved the final manuscript.
